# Conventional and Regulatory CD4^+^ T Cells That Share Identical TCRs Are Derived from Common Clones

**DOI:** 10.1371/journal.pone.0153705

**Published:** 2016-04-21

**Authors:** Kyle J. Wolf, Ryan O. Emerson, Jeanette Pingel, R. Mark Buller, Richard J. DiPaolo

**Affiliations:** 1 Department of Molecular Microbiology and Immunology, Saint Louis University, School of Medicine, Saint Louis, Missouri, United States of America; 2 Adaptive Biotechnologies Corporation, Seattle, Washington, United States of America; Jackson Laboratory, UNITED STATES

## Abstract

Results from studies comparing the diversity and specificity of the TCR repertoires expressed by conventional (Tconv) and regulatory (Treg) CD4^+^ T cell have varied depending on the experimental system employed. We developed a new model in which T cells express a single fixed TCRα chain, randomly rearranged endogenous TCRβ chains, and a Foxp3-GFP reporter. We purified CD4^+^Foxp3^-^ and CD4^+^Foxp3^+^ cells, then performed biased controlled multiplex PCR and high throughput sequencing of endogenous TCRβ chains. We identified >7,000 different TCRβ sequences in the periphery of 5 individual mice. On average, ~12% of TCR sequences were expressed by both conventional and regulatory populations within individual mice. The CD4^+^ T cells that expressed shared TCR sequences were present at higher frequencies compared to T cells expressing non-shared TCRs. Furthermore, nearly all (>90%) of the TCR sequences that were shared within mice were identical at the DNA sequence level, indicating that conventional and regulatory T cells that express shared TCRs are derived from common clones. Analysis of TCR repertoire overlap in the thymus reveals that a large proportion of Tconv and Treg sharing observed in the periphery is due to clonal expansion in the thymus. Together these data show that there are a limited number of TCR sequences shared between Tconv and Tregs. Also, Tconv and Tregs sharing identical TCRs are found at relatively high frequencies and are derived from common progenitors, of which a large portion are generated in the thymus.

## Introduction

CD4^+^Foxp3^+^ T regulatory cells (Tregs) are essential for preventing autoimmunity and regulating inflammation [1–6]. FoxP3, a transcription factor required for Treg development and function, is induced in CD4^+^ T cells in the thymus and in the periphery under various conditions [7, 8]. The role that T-cell receptor (TCR) specificity plays in the biology of Tregs is not completely understood. Evidence suggests that TCRs expressed by Tregs are specific for self-antigens [9–12]. If the TCRs expressed by Tregs are primarily specific for self-proteins, there should be limited overlap between the TCRs expressed by Tregs and CD4^+^FoxP3^-^ conventional T cells (Tconv), as the majority of Tconv are specific for non-self antigens. However, Tregs also play an important role in resolving and regulating the response to non-self antigens, indicating it is possible that Tregs are capable of expressing TCRs specific for non-self, and may therefore have greater overlap with the TCRs expressed by Tconv [13]. Reports on overlap in TCRs expressed by Tregs and Tconv have varied greatly, with estimates from 20% to 80% [14–16]. Because Tregs not only regulate immune responses to self-antigens, but also to microbes, parasites, vaccines, and allogeneic tissue [17–20], it is important to understand whether Tregs are specific for self-antigens and suppress responses to non-self in a bystander fashion, or whether Tregs are specific for non-self antigens, regulating immune responses to vaccines, pathogens, and transplanted tissue in an antigen specific manner.

In addition to TCR specificity, a major point of interest in Treg biology is where Tregs differentiate. Tregs are induced both in the thymus (“Thymic” Tregs), as well as in the periphery (“Peripheral” Tregs) [21, 22]. Currently there is no way to accurately distinguish between thymic Tregs and peripheral Tregs in the periphery. Because of this, the proportions of Tregs in the periphery that originate in the thymus and those that differentiate in the periphery are not well characterized.

To have an accurate and large-scale study of the TCRs expressed by Treg and Tconv populations, we developed a mouse model in which every T cell expresses an identical TCRα chain paired with a randomly rearranged endogenous TCRβ chain, and a Foxp3-IRES-GFP reporter. We refer to these as STAR mice (Single TCRα Retrogenic mice). Genomic DNA from purified Tconv and Treg cells from the spleen/lymph nodes (n = 5), and thymus (n = 3) of STAR mice was isolated, subjected to biased controlled multiplex PCR to amplify CDR3 regions of the TCRβ chains and high throughput sequencing to identify TCRβ sequences. Because all T cells in STAR mice express an identical TCRα chain, cells expressing identical TCRβ chain sequences also have identical TCR specificities.

On average, 12% of TCR sequences isolated from the spleen and lymph nodes were shared between Tconv and Tregs in individual mice. Shared sequences were found at higher frequencies compared to sequences that were not shared, and the 12% of the sequences that were shared comprised 46% of the CD4^+^ TCR repertoire. The thymus showed similar results as peripheral lymphoid tissues, where ~6% of TCR sequences were shared between Tconv and Treg populations within an individual mouse and shared sequences were found at high frequencies and accounted for ~40% of the CD4^+^ TCR repertoire. Additionally, we found that a significant portion of T cells within individual mice (but not between different mice) share identical TCR amino acid sequences also share identical DNA sequences. These data indicate that: 1) Tconv and Tregs share a limited number of TCRs, 2) shared TCRs are present at relatively high frequencies, and 3) Tconv and Tregs that share TCRs are derived from common progenitors that either lost or gained Foxp3 expression during clonal expansion. In addition, a substantial portion of the TCRs shared by Tconv and Treg populations in the periphery originate in the thymus.

## Methods and Materials

### Mice

TCRα^-/-^ and TCRβ^-/-^ mice and Foxp3-IRES-eGFP reporter mice [23] were obtained from Jackson Laboratories and maintained under specific pathogen free conditions. The TCRα^-/-^xTCRβ^+/-^xFoxP3eGFP mice were created by cross-breeding of TCRα^-/-^, TCRβ^-/-^, and FoxP3eGFP transgenic mice in our animal facility. All animal work has been conducted in accordance with the Guide for the Care and Use of Laboratory Animals of the National Institute of Health with approval from the Saint Louis University Institutional Animal Care and Use Committee.

### Retroviral transduction

pMIA DNA plasmid construct was created utilizing a modified pMIG plasmid as previously described [24]. Briefly, Phoenix A cells (PhxA) were transduced with 4:1 ratio of PMIAP2 plasmid:FuGene. Viral supernatant from PhxA cultures were filtered, and added to GP-E86 cells (with 8ug/mL polybrene). Cells were centrifuged at 3000rpm for 90min at room temperature. This process was repeated and the highest 10% of Ametrine expressing GP-E86 cells were sorted by FACS.

### Bone marrow derived hematopoietic stem cell generation

Bone marrow cells from 8 week old TCRα^-/-^xTCRβ^+/-^xFoxP3eGFP mice were treated as previously described [24]. Briefly, TCRα^-/-^xTCRβ^+/-^xFoxP3eGFP mice were injected with 0.15mg/g of body weight 5-Fluorouricil. 72hr later, bone marrow was removed from TCRα^-/-^xTCRβ^+/-^xFoxP3eGFP mice and stem cells were isolated. Bone marrow cells were retrovirally transduced as previously described [25].

TCRα^-/-^ mice on a C57BL/6J background were irradiated with 450rads. 3-5x10^6^ virally transduced bone marrow cells were adoptively transferred into each 8 week old TCRα^-/-^xTCRβ^+/-^xFoxP3eGFP recipient via tail vein injection.

### Single cell suspension preparation

Pooled cells from spleen and peripheral lymph nodes were enriched for CD4^+^ T cells with AutoMACS T cell enrichment magnetic bead separation. Cells were stained for CD4 and sorted via flow cytometry. Cells were gated on the Ametrine^+^ population. Gated cells were sorted into CD4^+^FoxP3eGFP^+^ population and CD4^+^FoxP3eGFP^-^ populations. Cells isolated from the thymus were CD8 depleted by AutoMACS T cell depletion and CD8^-^ cells were purified. Cells were stained for CD4 and CD8. Cells that were Ametrine^+^ were selected and sorted into CD4^+^CD8^-^FoxP3eGFP^+^ and CD4^+^CD8^-^FoxP3eGFP^-^ populations. Sorted cells were pelleted and sent to Adaptive Biotechnologies for amplification and deep sequencing of the TCRβ CDR3 region.

### DNA Sequencing

Genomic DNA from T cells was amplified using multiplexed primers targeting all V and J gene segments. TCRβ CDR3 regions were amplified and sequenced using ImmunoSEQ^™^. Synthetic templates mimicking natural V(D)J rearrangements were used to measure and correct amplification bias [26]. CDR3 segments were annotated according to the International ImMunoGeneTics collaboration [27, 28], identifying V, D, and J genes contributing to each rearrangement.

### FACS analysis

Eight weeks post adoptive transfer at approximately 16 weeks of age, flow cytometry was used to determine the reconstitution of the immune system. Cells were stained for CD4 and TCRβ. FoxP3 production was determined by endogenous GFP expression. Sorting of Tconv and Tregs was completed by gating on lymphocytes before gating specifically on Ametrine^+^ cells. Cells were stained for CD4 and ultimately sorted to Ametrine^+^xCD4^+^xFoxP3gfp^-^ Tconv cells or Ametrine^+^xCD4^+^xFoxP3gfp^+^ Tregs.

### Statistical analysis

Alignment of shared and non-shared TCRs was completed using ImmunoSeq software provided by Adaptive Biotechnologies. Graphical analyses were created using Graphpad Prism 5.0. Similarity indices measured with statistical estimation program EstimateS. Student’s t-test was used to determine statistical significance between TCR sequence overlaps between Tconv and Treg populations in STAR mice. The percent of sequence overlap between Tconv and Tregs was calculated taking into account the number of shared sequences to ensure shared sequences were not counted twice between Tconv and Treg populations.

## Results

### Single TCRα retrogenic (STAR) mice

**Fig 1:**
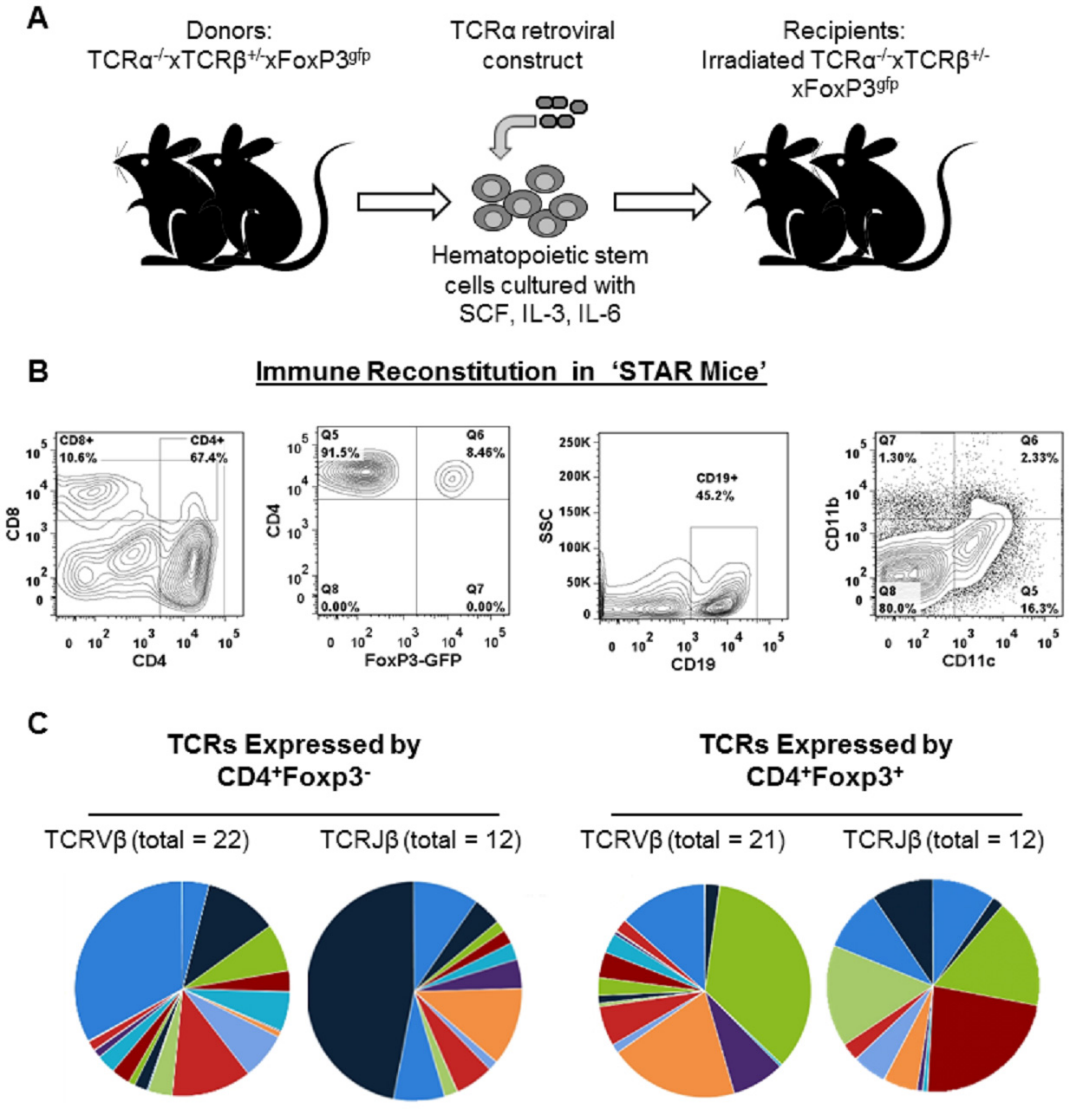
STAR mice display normal immune reconstitution with diverse TCRβ chain usage. A) Overview of method for making STAR mice. B) Representative flow cytometry plots showing CD4 vs CD8, CD4^+^Foxp3^-^ and CD4^+^Foxp3^+^, B cell, and APC cell distribution in STAR mice. C) Pie charts displaying the diversity of TCRVβ and TCRJβ usage in Tconv (left) and Tregs (right). Each color represents a different gene segment and the size of the section is proportional to the frequency of that gene segment.

To generate a new mouse model to compare TCRs expressed by Tconv and Tregs, we crossed commercially available strains of mice to generate TCRα^-/-^xTCRβ^+/-^xFoxP3eGFP mice. Hematopoietic stem cells from 8–10 week old TCRα^-/-^xTCRβ^+/-^xFoxP3eGFP mice were retrovirally transduced to express a single TCRα chain (TCRVα13) along with an Ametrine fluorescent reporter [24, 25]. Retrovirally transduced hematopoietic stem cells were then transferred into irradiated 8 week old TCRα^-/-^xTCRβ^+/-^xFoxP3eGFP mice. Eight weeks after transfer and reconstitution, the recipient mice were comprised of Ametrine^+^CD4^+^ T cells that stained positive for TCRβ, demonstrating that the retrovirally encoded TCRVα13 chain restored T cell development (Fig 1A). Since mice are deficient for TCRα with the exception of the retrovirally implanted TCRVα13, there is no possibility for leaky expression of endogenous TCRα chains. 100% of all αβ T cells in STAR mice express TCRVα13 and a single endogenously rearranged TCRβ chain.

STAR mice developed the expected frequencies of CD4^+^Foxp3^-^ conventional T cells (Tconv), CD4^+^Foxp3^+^ regulatory (Tregs) T cells, CD8^+^ T cells, CD19^+^ B cells, and antigen presenting cells (Fig 1B). STAR mice appear healthy and have no indication of autoimmunity, indicating the Treg cells are functional. Previous studies utilizing retrogenic mice have also demonstrated that retrogenic models produce healthy mice that reconstitute a “normal” immune system akin to what is seen in traditional bone marrow transfer models [25, 29–32].

Next, we examined the diversity of the TCRβ chains expressed by CD4^+^ T cells from STAR mice. CD4^+^ T cells were sorted into FoxP3-GFP^+^ Treg and Fox-GFPP3^-^ Tconv populations by FACS. After sorting, Tconv and Treg cells were >99% purity and cross-contamination between purified Tconv and Tregs was undetectable (data not shown). On average, >1x10^6^ Tconv and >1.5x10^5^ Tregs were collected and sequenced from each mouse. TCRβ chains were amplified with multiplex biased controlled primers that amplified every combination of TCRVβ and TCRJβ gene segment rearrangements, and subjected to high-throughput sequencing of all TCRVβ-Jβ rearrangements. From 1.2x10^6^ purified CD4^+^ T cells from a single mouse, we identified 1,066 and 637 different TCRβ chain sequences in Tconv and Treg populations respectively. TCR sequences that were identified <5 times in a sample were removed from the individual sample to decrease any chance of false-positive sequences occurring from sequencing errors from entering the data set. The TCRβ chains expressed by CD4^+^ T cells from STAR mice were diverse, and included 22 different TCRVβ gene segments in Tconv and 21 TCRVβ segments in Tregs. Both Tconv and Tregs expressed 12 different TCRJβ gene segments (Fig 1C). Admittedly, the use of a single TCRα chain to reconstitute the entirety of the αβ T cell repertoire will produce a T cell population with restricted TCRβ diversity compared to wild type. We have calculated that our mice have a TCRβ diversity approximately 20–50% that of wild type mice (data not shown). To summarize, STAR mice are single TCRα chain retrogenic mice, they contain the expected frequencies of CD4+Foxp3-Tconv and CD4^+^Foxp3^+^Tregs, and these cells express a diverse array of different TCRβ chains. These mice were developed to provide an accurate and in depth comparison of the TCRs expressed by Tconv and Tregs.

### Comparing TCRs expressed by Tconv and Tregs in STAR mice

**Fig 2:**
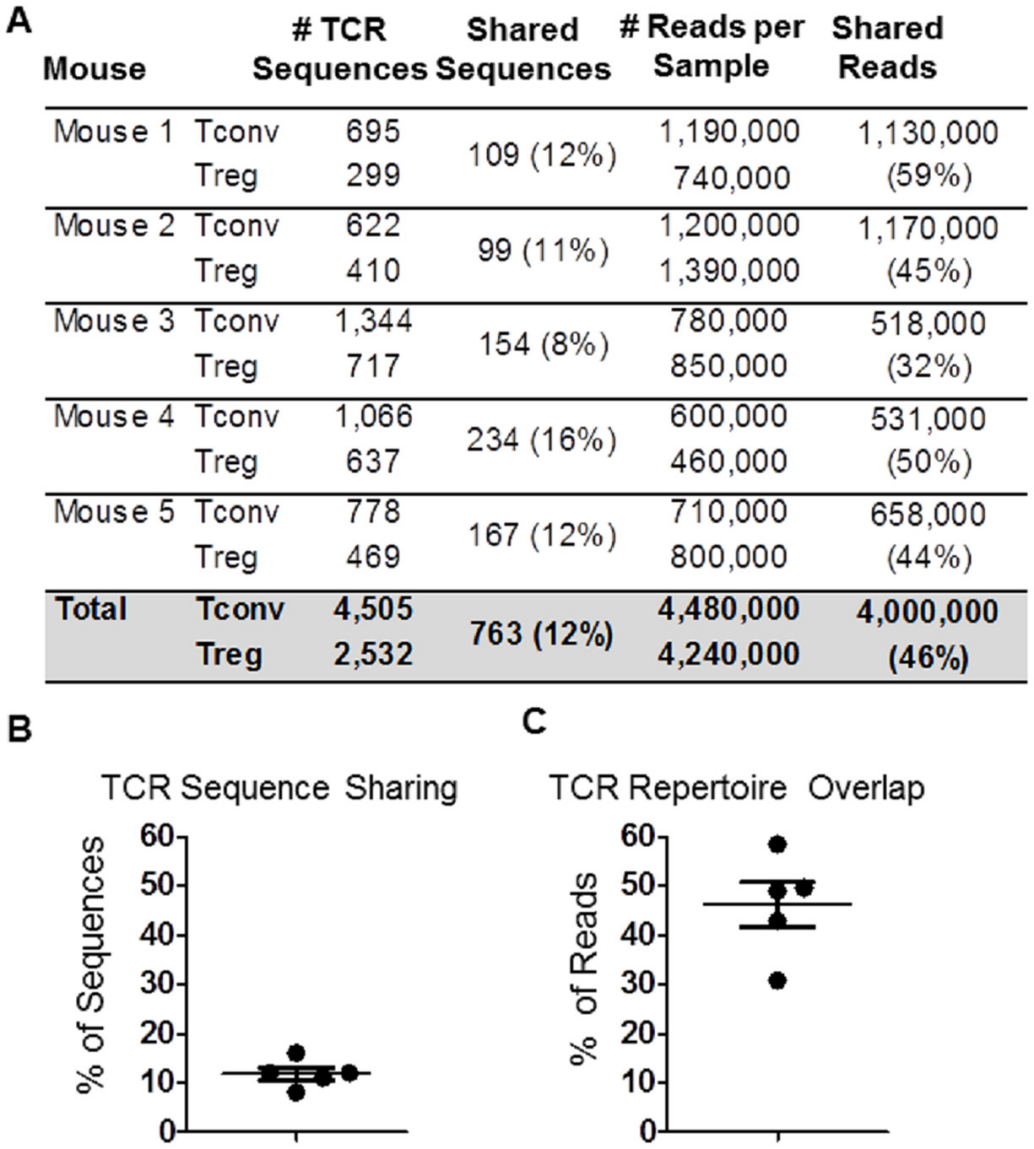
TCR sharing by CD4^+^Foxp3^-^ (Tconv) and CD4^+^Foxp3^+^ (Treg) cells. A) Tabulated results displaying the number of individual TCRβ chain amino acid sequences identified in each mouse, and the number and percent of TCRs shared between Tconv and Tregs within each mouse. B-C) Graphical representations of the percentage of TCR sequences shared (left) and TCR repertoire (right) overlap between Tconv and Tregs within individual STAR mice, after accounting for the frequencies of TCR sequences (n = 5, Mean ± SE).

Next, TCRβ sequences expressed by Tconv and Tregs from STAR mice were analyzed. Briefly, CD4^+^Foxp3^-^ (GFP^-^) and CD4^+^Foxp3^+^ (GFP^+^) T cells were sorted from the spleens and lymph nodes of 5 individual STAR mice. Genomic DNA was isolated from each sample, which was then amplified and subjected to sequencing. This resulted in an average of 4x10^5^-1.5x10^6^ sequence reads per sample, and more than 7,000 individual TCRβ chain amino acid sequences identified; 4,505 from Tconv cells, and 2,532 from Tregs. Of these, 763 (mean ± SE = 12.2% ± 1.4 between all 5 mice) TCRβ sequences were found in both populations (Fig 2A and 2B). These data demonstrate that a limited proportion of TCR sequences in an individual mouse (~12%) are expressed by both the Tconv and Treg populations.

To determine how shared TCRs were distributed, we examined the frequencies at which each TCR was measured. The number of times a TCRβ chain sequence appears in a dataset (reads) is proportional to the frequency of CD4^+^ Tconv or Treg cells in the population that were expressing that TCR. For example, if the sequences of “TCR A” was present 1,000 times in the dataset and “TCR B” present 100 times, the frequency of CD4^+^ cells expressing TCR A in the sorted population was 10-fold higher than the frequency of cells expressing TCR B. An analysis of the relative abundance of TCR sequences that were either shared or not shared between Tconv and Treg samples was performed. When the relative frequency of each individual TCR sequence was taken into account, the 12% of individual sequences that were shared between Tconv and Tregs were found at such high frequencies that they accounted for 46% of all of the reads in the dataset (Fig 2A and 2C, mean ± SE = 46% ± 4.5). In contrast, the 88% of the TCR sequences that were not shared between Tconv and Tregs only accounted for 54% of the total number of sequences in the datasets. These data demonstrate that although the proportion of TCRs shared between Tconv and Tregs are limited (12%), shared sequences are found at relatively high frequencies in the repertoires compared to non-shared sequences.

**Fig 3:**
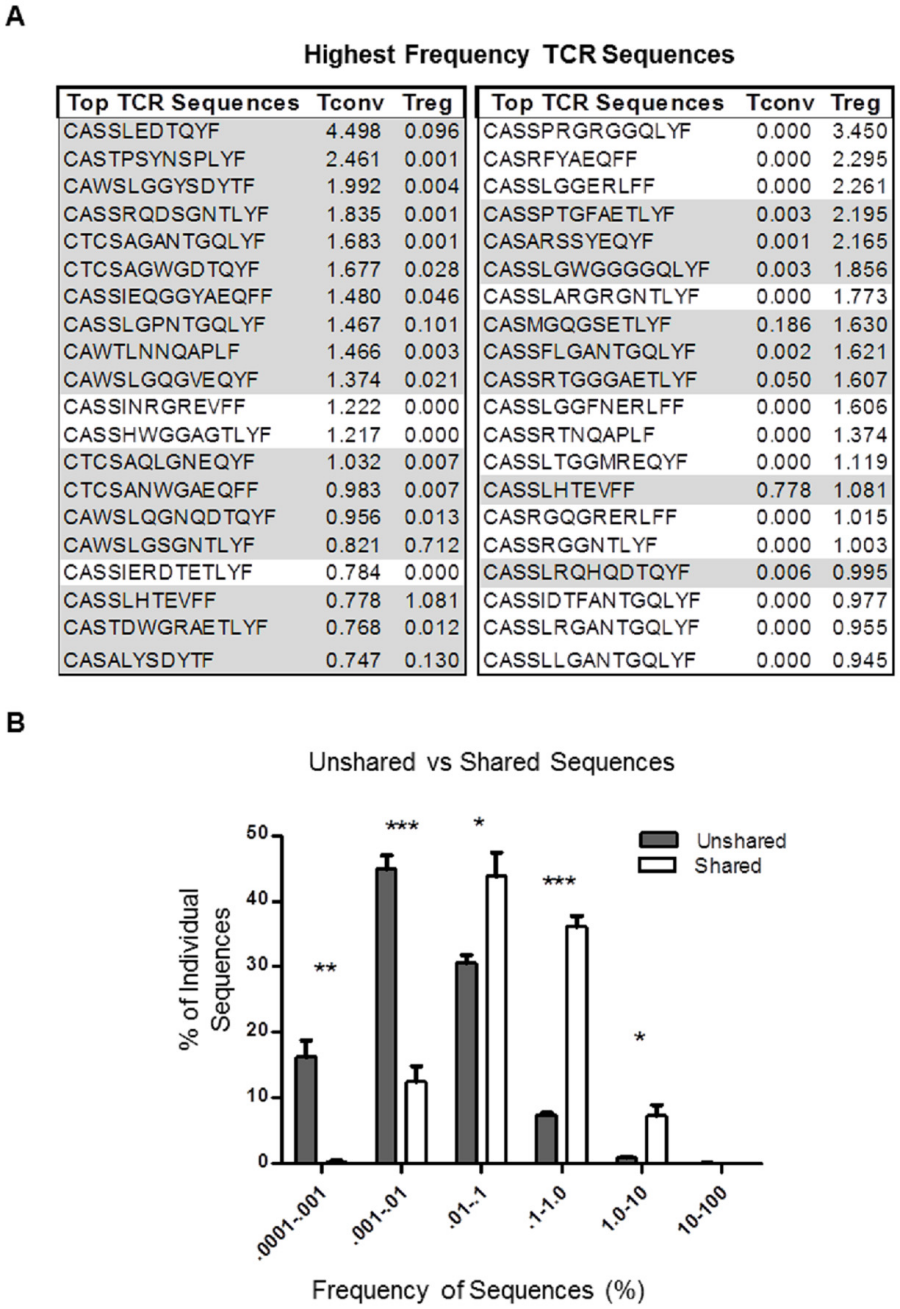
Shared sequences are found at higher frequencies in peripheral Tconv and Tregs. A) The sequences and frequencies of the most abundant TCRs in Tconv and Tregs and whether they are shared (gray) or are not shared (uncolored). B) Sequences that are not shared (grey) are found at significantly lower frequencies, while shared sequences (white) are found at significantly higher frequencies in the repertoires. Frequencies are measured as percentage of entire repertoire. Statistical significance was determined using the student’s t-test (n = 5, Mean ± SE, * p<.05, ** p<.01, *** p<0.0001).

To follow up on this observation, we analyzed the 20 most frequent TCR sequences from both the Tconv and Treg repertoires of all 5 mice. Of the 20 most frequent TCR sequences identified in the Tconv populations, 17 (85%) were also identified in the Treg repertoire of the same mouse. Eight of the 20 most frequent TCR sequences identified in the Treg TCR repertoire were also identified in the Tconv repertoire (Fig 3A). When analyzed within individual mice, a significantly larger percentage of shared TCRs are found at higher frequencies than non-shared sequences. For example, only ~40% of non-shared TCRs in Tconv and Treg populations are found ≥0.01% of the repertoire compared to >85% of the shared TCR sequences (Fig 3B). To summarize, the relatively small number of TCR sequences shared by Tconv and Treg cells (12%) are found at relatively high frequencies and account for a large percentage (46%) of the CD4^+^ TCR repertoire.

### Large portion of TCR repertoire overlap in the periphery originates in the thymus

**Fig 4:**
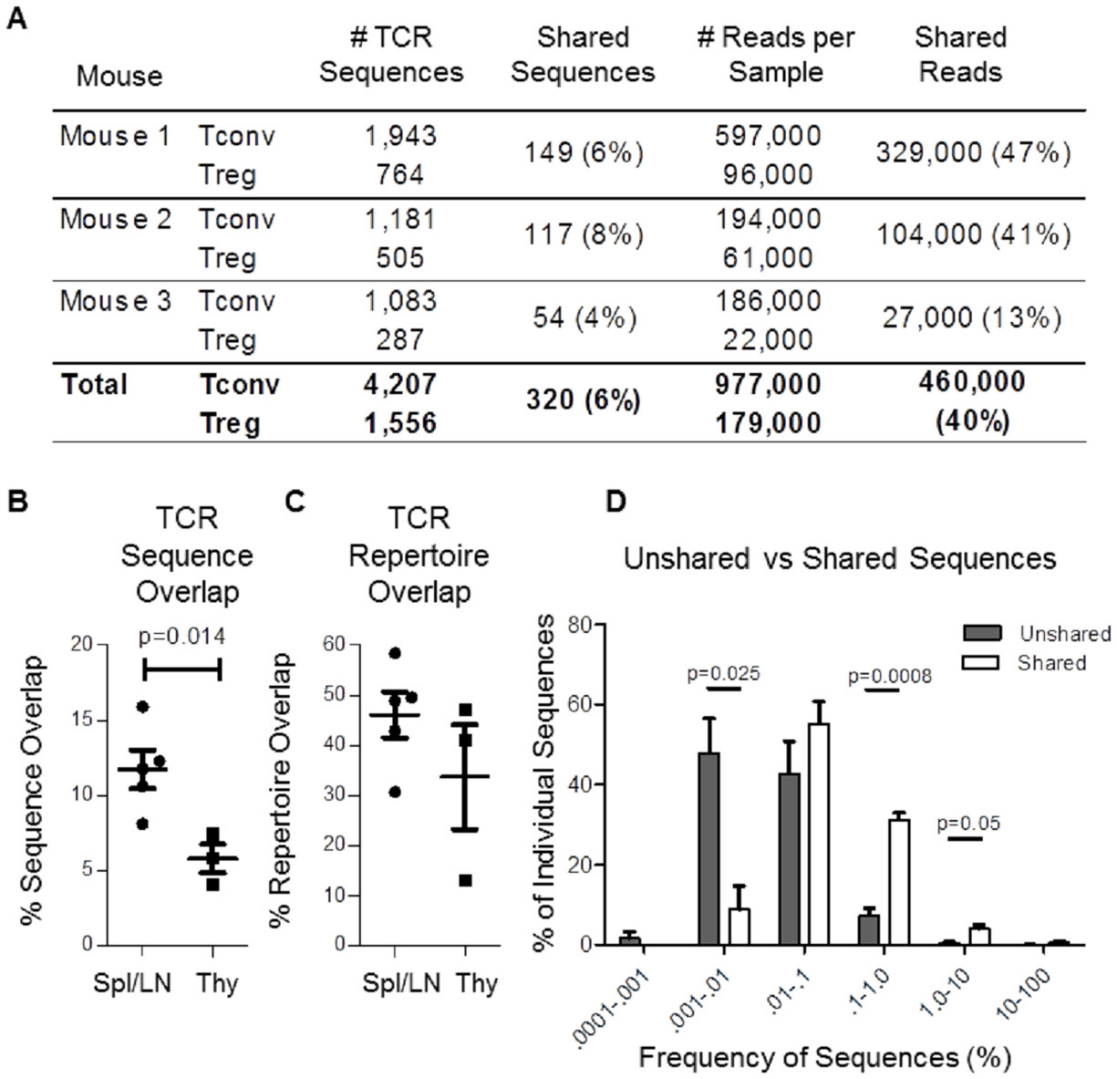
Limited sharing of high frequency TCRs in the thymus. A) Tabulated results displaying the number of individual TCRβ chain amino acid sequences identified in the thymus of each mouse, and the number and percent of TCRs shared between Tconv and Tregs within each mouse. B) Comparison of the percentage of individual sequences shared by Tconv and Tregs in the spleen/lymph node and thymus of STAR mice. C) Comparison of the percent of all analyzed CD4^+^ T cells with TCR sequences that are shared by Tconv and Treg populations within the periphery or the thymus. D) Proportions of shared (white) or unshared (gray) TCR sequences found within particular frequency intervals in the thymus of STAR mice. Frequencies are measured as percentage of the entire CD4^+^ repertoire. Statistical significance was determined using the student’s t-test (n = 3, Mean ± SE, * p<.05, ** p<.01, *** p<0.0001).

Tconv and Tregs that share identical TCRs may originate in the thymus, periphery, or both. In order to determine if Tconv and Treg populations that have identical TCRβ sequences are expanded in the thymus, we performed a similar analysis of TCR sequences expressed by Tconv and Treg isolated from the thymus. A total of 5,443 individual TCR sequences were identified from CD4^+^ T cells in the thymii of 3 individual STAR mice. On average, ~6% of individual TCRs were found in both Tconv and Treg populations (Fig 4A). The number of overlapping sequences in the thymus was approximately half of what is observed in the periphery (Fig 4B). However, like in the periphery, shared TCR sequences in the thymus were also found at relatively high frequencies compared to TCR sequences that were not shared, and the proportion of the TCR repertoire shared between Tconv and Treg populations was similar in the thymus and the periphery (Fig 4C and 4D).

### Tconv and Tregs with shared TCRs are derived from common progenitors

Redundancy in the genetic code makes it possible for proteins that have identical amino acid sequence to have different DNA sequences. Up to this point, we have compared TCR sequences between Tconv and Tregs at the amino acid level, as identical amino acids encode for identical specificities. Comparing TCR sequences at the DNA level provides additional information. A Tconv and Treg that express a TCR that is identical at the amino acid level but not the DNA level cannot be derived from a common progenitor clone. However, a Tconv and Treg that express a TCR that is identical at both the amino acid level and the DNA level are likely to be derived from a common progenitor clone.

**Fig 5:**
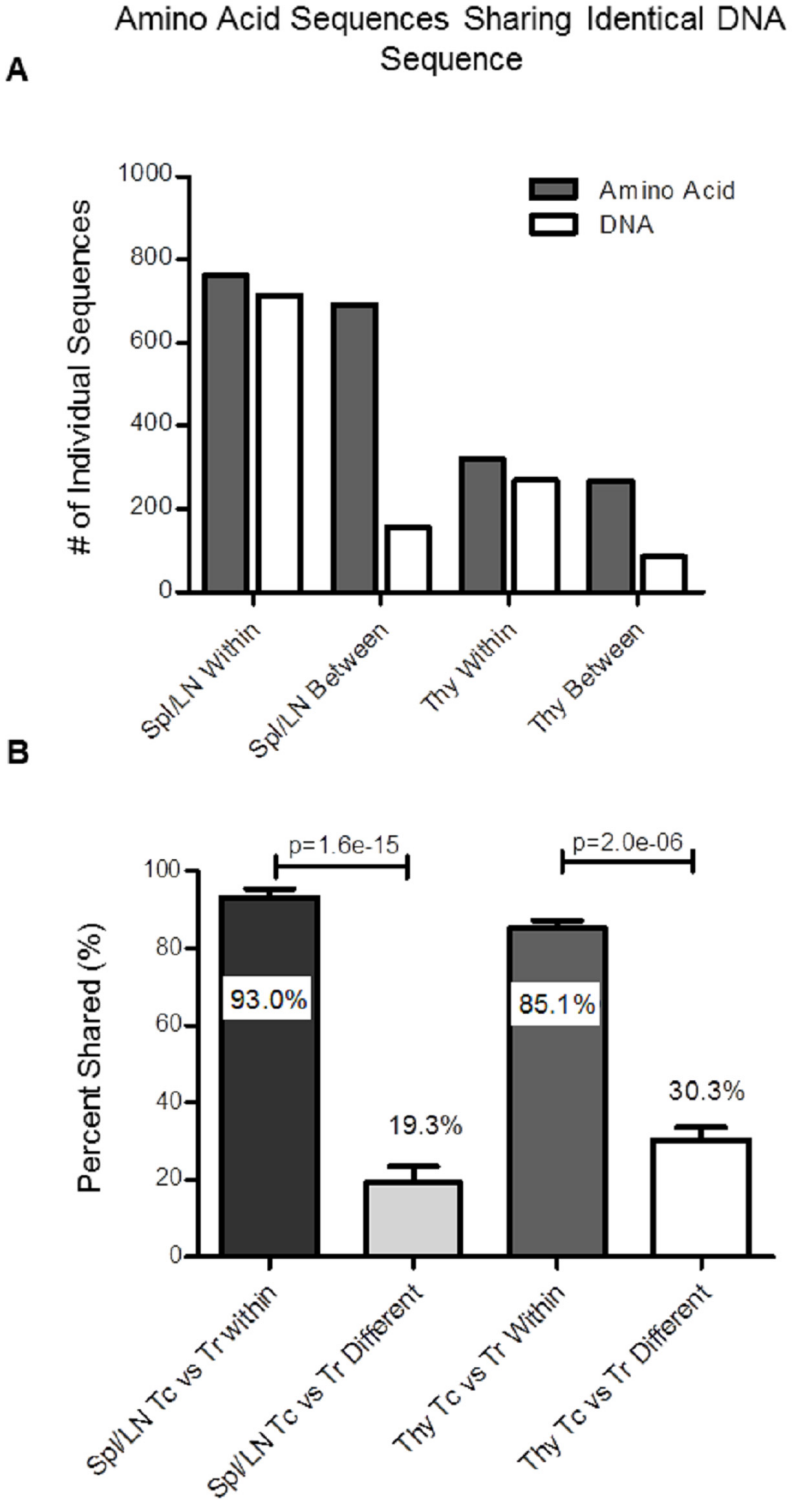
Analysis of amino acid and DNA TCRβ sequence sharing in the thymus and periphery of STAR mice. A) Comparison of the total number of TCRβ sequences shared between Tconv and Treg populations at the amino acid level (gray) and the number of shared amino acid sequences also sharing an identical nucleotide sequence (white) within individual mice, or between mice, in the periphery (Spl/LN, left) and thymus (Thy, right). B) Percentage of TCR sequences shared at the amino acid level that also share identical nucleotide sequences by Tconv and Treg populations within an individual mouse or between individual mice in the periphery (n = 5) and thymus (n = 3) of STAR mice. Statistical significance was determined using Fisher’s exact test and student’s t-test (Mean ± SE, *** p<0.0001).

To determine whether Tconv and Tregs that share TCR amino acid sequences were derived from common or independent precursors, we analyzed sequences that were shared among Tconv and Tregs in different mice, which cannot be derived from common clones, and TCR sequences shared by Tconv and Tregs within individual mice, which may or may not be derived from common clones, at both the amino acid and DNA levels. In total, 692 different TCRβ chain sequences at the amino acid level were found to be shared between Tconv and Tregs isolated from the periphery of any 2 given mice for all 5 individual mice analyzed (Tconv repertoires from mouse 1 are compared individually to Treg repertoires from mouse 2, mouse 3, mouse 4 and mouse 5, etc.). Of those 692 sequences, only 155 (22%) were also identical at the DNA level. In contrast, of the 762 different TCRβ chain sequences were shared between Tconv and Treg populations within individual mice, and nearly all, 713 (94%) were also identical at the DNA sequence level (mean ± SE: 93% ± 2.5% within mice, and 22% +/- 4.9% between mice, P <1x10^-11^). Similarly, 268 amino acid sequences were shared between Tconv and Treg populations between the thymii of any 2 given mice from the 3 individual mice analyzed. Of those 268 sequences only 85 (31.7%) shared identical nucleotide sequences. Within individual mice, of the 320 TCR amino acid sequences shared by Tconv and Treg populations, 269 (84%) also displayed identical TCRβ gene sequences (Fig 5A and 5B). It is important to note that all observations put forth in this manuscript were analyzed at both the amino acid and nucleotide level, from which we were unable to measure any observable differences when analyzing the TCR repertoire overlap at the amino acid and nucleotide level between Tconv and Tregs (data not shown). These data demonstrate that the majority of Tconv and Treg cells that share TCR sequences in an individual are likely to be derived from common progenitors. This could occur if a subset of Tconv acquired Foxp3 expression during expansion, or if a subset of Tregs lost Foxp3 expression during expansion.

In summary, we display that the proportion of TCRs shared by Tconv and Tregs are slightly reduced in the thymus compared to the periphery in STAR mice. However, shared sequences remain present at higher frequencies compared to unshared sequences. In addition, a large proportion of the TCRs identified in both Tconv and Tregs also share identical nucleotide sequences, indicating that, similar to the periphery, Tconv and Tregs expressing identical TCRs are derived from common progenitors in the thymus.

## Discussion

A new mouse strain (STAR mice) and deep sequencing technology allowed for the analyses of Tconv and Treg TCRβ repertoires in 5 individual mice. As all T cells expressed the exact same TCRα chain, finding identical TCRβ chain sequences accurately identified CD4^+^ T cells with the same TCR specificities. The limited overlap in TCR sequences (12%), suggests that Tregs and Tconv express distinct TCR repertoires, and supports models that propose that Tregs express self-reactive TCRs and Tconv express non-self-reactive TCRs, with limited overlap [33, 34].

Although the numbers of shared TCR sequences were limited, the shared sequences were found at relatively high frequencies compared to those that were not shared, and accounted for a large portion of the CD4^+^ T cell repertoires. These results are supported by observations made by Wong et. al. 2007, where Treg and Tconv TCR repertoires were reported to have a limited amount of identical sequences that accounted for a significant proportion (0.42) of the repertoire [35]. When analyzing high frequency TCRs, Pachnolczyk et. al. 2007 also observed that high frequency TCRs were commonly shared between Tconv and Tregs, concluding that TCRs in Treg repertoires are predominantly specific for non-self [14]. Our findings, which include both high and low frequency TCRs, do not support the concept that the majority of TCRs are shared by Tconv and Tregs.

The data presented here indicate that Tconv and Tregs that share a TCR sequence, within an individual, are derived from common clones. This is likely to be the result of one or more scenarios: 1) A subset of self-reactive TCRs expressed by both Tconv and Tregs expand in response to self-antigen [14]; 2) During clonal expansion, a small percentage of differentiating CD4^+^FoxP3^-^ T cells acquire FoxP3 expression (example: peripheral induction of Tregs to commensal microbiota) [7, 36]; and 3) Foxp3 expression was lost by a subset of Tregs (i.e. ex-Tregs), which then appeared in the Tconv repertoire [37, 38]. While it is appreciated Tregs can develop from naïve CD4^+^ T cells in the periphery (pTregs), the origin of peripherally induced Tregs has been a matter of debate, with evidence that they either arise independently of effector CD4^+^ T cell expansion or as a subset of rapidly proliferating differentiated effector T cell populations [14, 16]. While the presence of peripherally induced Tregs has been established, there is no reliable method to distinguish between thymic and peripherally induced Tregs.

It is worth noting that with most shared sequences, sequence distribution is heavily skewed towards either Tconv or Treg populations, but rarely evenly distributed. This can be seen in Fig 3, where even though 85% of the highest frequency TCR sequences in Tconv populations are shared with Tregs, the frequencies of those sequences in Tregs are low. This observation supports a model in which, a small percentage of CD4^+^FoxP3^-^ T cells acquire FoxP3 expression during clonal expansion in the periphery, or alternatively, only a small subset of T cells are induced to express FoxP3 in the thymus [7, 36].

After examining the Tconv/Treg TCR overlap in the thymus of STAR mice, we came to the conclusion that a relatively large portion of the TCR sharing between Tconv and Tregs in the periphery originates in the thymus. Most likely, during expansion and selection, a subset of a T cell clone are exposed to self-peptide in the context of MHC-II for which the TCR has relatively high binding affinity. Instead of being negatively selected, a portion of these cells are instructed to express FoxP3 and become Tregs. This mechanism had been originally characterized in a publication by Lee et. al. as a “Treg Niche” that was implicated to occur during T cell selection in the thymus [11]. While much of the Tconv/Treg TCR sharing in the periphery can be explained by T cell expansion in the thymus, there still remains a portion of the TCRs in the periphery that are shared by Tconv and Tregs that are likely the result of peripheral activation of naïve CD4^+^ which then expanded and differentiate.

Since the analysis of the TCRs present in the thymus can only determine the TCRs on T cells currently undergoing selection, determining the proportion of the periphery TCR overlap that originates in the thymus is not possible. Thymic analysis and peripheral analysis was completed on different mice. The thymic TCR repertoire changes constantly as new T cells pass selection, expand, and exit the thymus. There is no evidence that T cells remain resident in the thymus after selection. Following this logic, there is little reason to suspect that T cells undergoing clonal expansion in the thymus would be readily identified in the periphery or vice versa, no reason to expect shared TCR sequences in the periphery to remain detectable in the thymus. However, we can determine that even though the percentage of individual TCR sequences in the thymus is less than half of what is found in the periphery, shared sequences are consistently found at significantly higher frequency than unshared sequences.

Ultimately, the data presented here cannot say for certain whether expanding CD4^+^Foxp3^-^ T cells acquired Foxp3 expression, or whether expanding CD4^+^Foxp3^+^ T cells lost Foxp3 expression. Though the observation that shared TCRs in the Tconv populations are predominately found at high frequencies, while the frequencies of shared and unshared TCRs in Tregs are more equally distributed in both the periphery and thymus, leads us to favor a model wherein naïve CD4^+^ T cells expand and differentiate into effector T cells, and from those differentiating cells, a small subset gain expression of FoxP3 and acquire regulatory function. In the thymus, this may be due to expanding clones being pushed toward Tconv and Treg cell fates after exposure to different self-antigen; in the periphery, the differentiation may be caused by the local cytokine milieu. From these data, we are able to conclude that a small fraction of TCRs shared by Tconv and Treg populations occupy 46% of the peripheral CD4^+^ TCR repertoire, and that the vast majority of Tconv and Tregs that express identical TCRs in an individual are derived from common precursors. In addition, we show that a substantial proportion of TCR sharing by Tconv and Tregs in the periphery originates during clonal expansion in the thymus.
